# Mechanistic models of microbial community metabolism

**DOI:** 10.1039/d0mo00154f

**Published:** 2021-03-23

**Authors:** Lillian R. Dillard, Dawson D. Payne, Jason A. Papin

**Affiliations:** Department of Biochemistry and Molecular Genetics, University of Virginia Charlottesville VA 22908 USA papin@virginia.edu; Department of Biomedical Engineering, University of Virginia Box 800759, Health System Charlottesville VA 22908 USA

## Abstract

Microbial communities affect many facets of human health and well-being. Naturally occurring bacteria, whether in nature or the human body, rarely exist in isolation. A deeper understanding of the metabolic functions of these communities is now possible with emerging computational models. In this review, we summarize frameworks for constructing mechanistic models of microbial community metabolism and discuss available algorithms for model analysis. We highlight essential decision points that greatly influence algorithm selection, as well as model analysis. Polymicrobial metabolic models can be utilized to gain insights into host-pathogen interactions, bacterial engineering, and many more translational applications.

## Abstract 2

Microbial communities affect many facets of human health and well-being. Naturally occurring bacteria, whether in nature or the human body, rarely exist in isolation.

## Graphical abstract

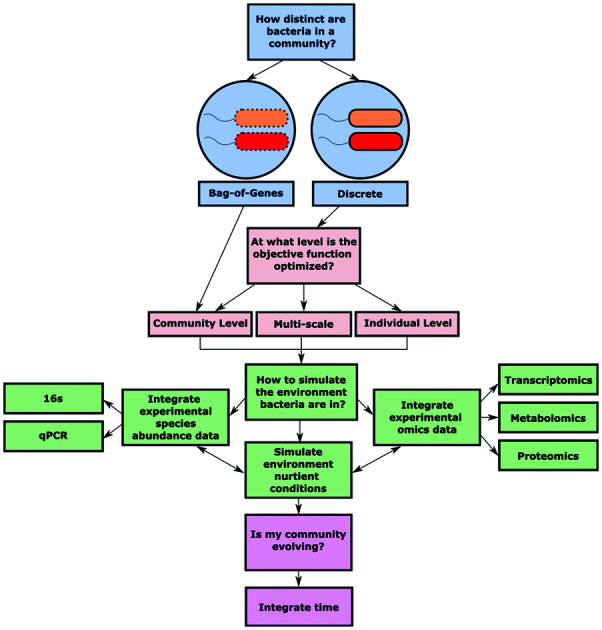

## Introduction

Bacterial communities have an ever-expanding impact on both the global ecosystem, through nutrient cycling, and society, through the health benefits and burdens they cause. In the human gut microbiota, bacteria type and abundance have direct implications for nutrient absorption, age-associated diseases such as Alzheimer's, and susceptibility to gastrointestinal infection. Without the microbiome, up to 30% of ingested energy would be lost *via* excrement because the human body can not completely digest consumed food on its own.^1^ The microbiome plays a role in host immunity by modulating mucus levels in the human gut, directly impacting resistance to invading pathogens.^2^ Identifying metabolic biomarkers of disease has allowed for improved therapeutic development. Bacteria also play a key role in a variety of industries. Engineered *Escherichia coli* are used to produce commercial quantities of insulin and microbial inoculants are used in soil to sequester carbon from the atmosphere.^3,4^ Knowledge of the bacterial metabolic shifts that result in product synthesis has been utilized to create effective metabolic systems in these industries. In the future, a better understanding of bacterial community metabolism will inform decisions to address questions in industry, health care, and basic science.

There is little known regarding microbial metabolism at the community level. Bacteria behave differently when in natural environments inhabited by multiple species compared to in isolation, which is typical in most laboratory experiments. Polymicrobial community interactions are governed by a set of poorly understood rules that encapsulate the tension between competition, genetic parsimony, and energy conservation. Experimental studies of these interactions often involve isolating the bacteria of interest and then co-culturing with other bacterial species to evaluate the community effects. This strategy presents a significant barrier for the field, as many bacteria are difficult or impossible to culture in a laboratory setting. Computational modeling can help to overcome this hurdle, enabling the study of these interactions *in silico*. Additionally, the high throughput capability of modeling is conducive to exploring community systems that would be impossible to systematically explore experimentally, allowing for the beginning of mechanistic understanding of bacterial metabolism in community settings.

Genome-scale network reconstructions (GENREs) and genome-scale models (GEMs) are powerful tools for studying bacterial community metabolism. GENREs function primarily as a repository for the biochemical reactions an organism is capable of executing. GEMs are derived from GENREs that have undergone both gap-filling and manual curation in order to achieve a high level of predicted metabolic output accuracy. GEMs can be utilized for computational simulations to further biological investigations and discoveries. By integrating multiple GEMs of various bacteria into one simulated community, the metabolic interactions of the polymicrobial community can be analyzed using Flux Balance Analysis (FBA) and related modeling methods. FBA-based simulations can provide information on what genes, metabolic pathways, and metabolites are utilized by community members in simulated environments. Integrating omic data into the model provides a layer of physiological insight in relation to gene transcription, metabolite usage, and protein synthesis, depending on the data used. Through understanding community metabolism we can better predict bacterial evolution, host–pathogen interactions, and improve bacterial engineering.

In this review we discuss FBA-based algorithms that allow for the *in silico* simulation of polymicrobial communities and the metabolic interactions that occur between community members. Through an understanding of GEM construction and curation we gain insight into how additional omic data integration can add context to these metabolic models. After a single bacterial GEM has been constructed we can tackle the question of what kind of bacterial community we want to model, as well as what kind of analysis we want to perform, and the algorithms that can aid in this process. Polymicrobial metabolic models allow for a deeper understanding of both the individuals within a community and the function of a community as a whole. With this knowledge we gain the ability to better modulate these behaviors in order to produce desirable outcomes.

## Metabolic model construction

GENREs are high quality repositories of information that synthesize biochemical knowledge into a convenient, computationally interpretable format.^5,6^ GENREs can capture diverse information including stoichiometric mappings of substrates to products and the enzymes that catalyze these chemical transformations. With the use of many analytical methods that have emerged, these complex networks can enable the investigation of numerous metabolic states.^7,8^

**Fig. 1:**
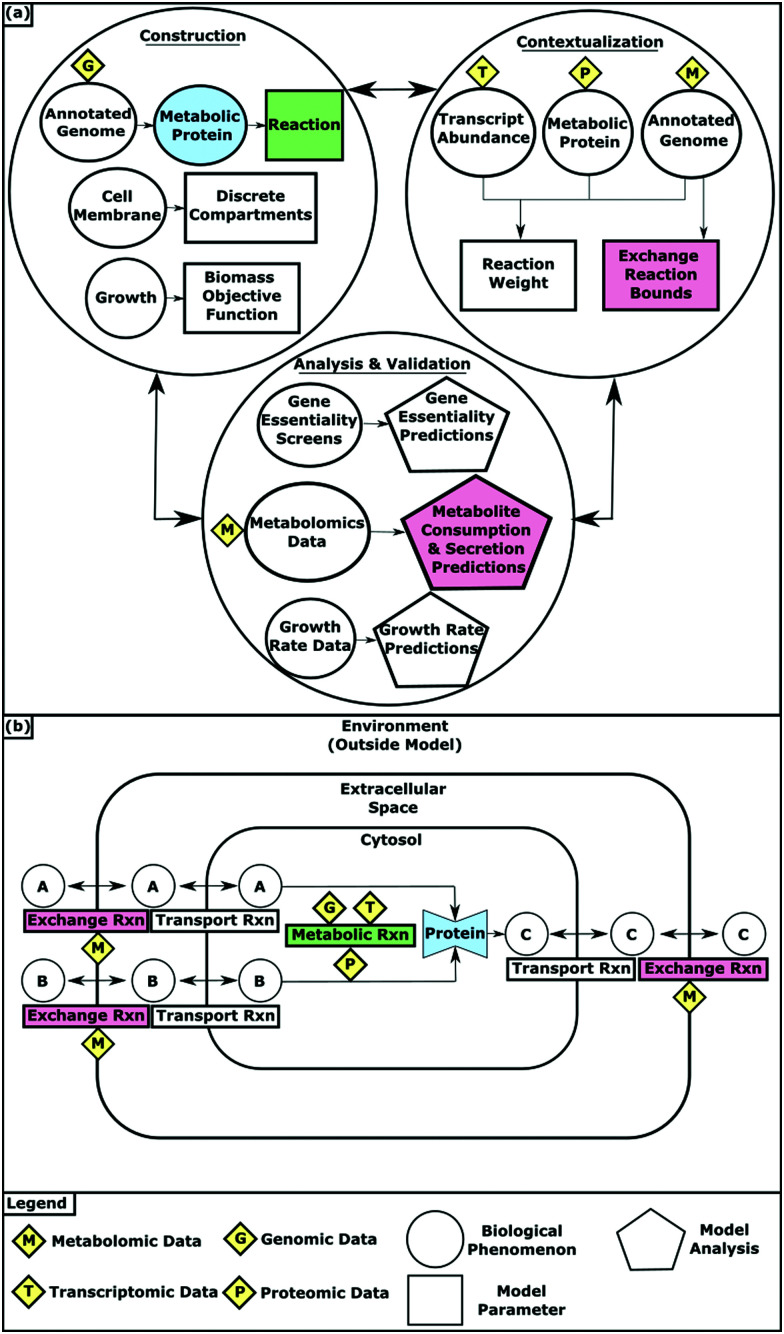
(a) Multi-omic data integration into GEMs during model construction, contextualization, validation and analysis. (b) How GEMs simulate the physiological conditions of an organism.

Network construction begins with the annotated genome (Fig. 1). The genes an organism contains determines the proteins it can synthesize and therefore the metabolic reactions it can catalyze. These associations are stored as gene–protein-reaction (GPR) relationships with the reactants and products of each reaction catalogued in a stoichiometric matrix. Reaction bounds capture the kinetic constraints and reversibility of reactions by dictating the amount and direction of flux that an individual reaction can carry. Metabolites in the model are assigned to compartments that simulate biologically discrete spaces such as the cytosol and the extracellular space. Exchange reactions are required to “place” metabolites within the confines of the model. Exchange reactions function by introducing and removing metabolites from the extracellular space, allowing those designated metabolites to then be accessible for transport into the modeled organism. Transport reactions allow metabolites to flow between the extracellular and cytosolic compartments. As the metabolic network takes shape, objective functions (OFs) that represent metabolic goals are added to the model to enable the interrogation of biological processes. For example, biomass synthesis OFs account for all of the metabolic components that must be synthesized for growth and allow for growth simulations. Draft models containing all of these components can be automatically created using tools such as ModelSEED and CarveMe.^9,10^

### Curation

Initially constructed models often contain key inconsistencies when compared to physiological data, and those inconsistencies require curation to resolve. Curation refers to a broad range of activities aimed at improving the overall quality of the model. Models are curated to ensure that they are able to carry flux through the biomass synthesis OF. Gap filling algorithms can be used to ensure model growth by adding reactions from established databases.^11,12^ While these automated curation algorithms circumvent the labor of manual curation, they can reduce model quality by adding reactions without strong biological evidence.

Another common target of curation is the resolution of energy generating cycles. These cycles are thermodynamically infeasible loops that are capable of charging electron carriers (such as ATP) without metabolite consumption.^13^ Unresolved loops can have effects that permeate through the model, leading to issues such as inflated growth yield predictions. These cycles can be caused by imbalanced reactions, reaction reversibility issues, or a multitude of other model errors. Loops are typically investigated with the aid of algorithms, but ultimately resolved through manual curation.^13,14^

While recently there have been significant gains in the automation of the curation process, the best models in the field are often the result of extensive manual curation. New versions of the widely utilized *Escherichia coli* and human metabolic models have been released following multiple rounds of curation.^15,16^ Manual curation is often an arduous task due to the wide variety of curation points traversing carbon source utilization predictions, reaction validity, and annotation consistency. However, the curation process results in a deeper understanding of organismal metabolism, often a reconciliation of conflicting literature, as well as a higher quality model.

### Contextualization

Frequently, with known constraints on reaction fluxes, there remains a large set of potential metabolic states of the network. Experimental data can be used to contextualize the model, trimming possible states to only those that are most biologically accurate in a given environment or experimental condition.

Transcriptomic and proteomic data provide evidence for what metabolic pathways are utilized by an organism at a given point in time or in a particular environmental condition. Transcriptomic data indicates which genes are expressed and methods often use that expression state as a surrogate for which reactions are available to the network. Protein abundance data offers more direct evidence for reactions available to the network based on what proteins are present. However, transcriptomic data is often more comprehensive and more commonly used for contextualization since it is more readily available. Several algorithms have been developed to integrate transcriptomic and proteomic data with metabolic models.^17–20^ These methods typically convert gene expression or protein abundance levels to reaction weights that represent the likelihood that individual reactions are being utilized by the organism. By applying these weights to the model, the predicted metabolic state can be guided towards what is observed experimentally.

Metabolomic profiles of supernatants from growth culture experiments provide evidence for the metabolites an organism consumes and produces as it grows. This data can be integrated as bounds on the exchange reactions that force the model to mimic the uptake and secretion of metabolites that were observed experimentally. It can also be analyzed as evidence for what metabolic pathways are being utilized and therefore integrated into the model as reaction weights.^21^

### Analysis

When assessing the quality of a metabolic model there are two main types of tests that are performed; those that measure accuracy of predictions about the corresponding biology and others that evaluate model standardization. Efforts to assess accuracy of the biological predictions involve the comparison of model predictions to experimental data. The growth of an organism in a defined media is both relatively simple to measure experimentally and a prediction that such metabolic network models can readily generate, offering a direct comparison to evaluate accuracy. This comparison allows for the absence (or erroneous presence) of metabolic pathways to be identified as points for curation. However, while this assessment of growth is a convenient test for easily cultured bacteria, it cannot be utilized for others that are difficult to grow in isolation in a laboratory setting. There are several other types of biological predictions frequently used to assess model quality as described elsewhere.^22,23^ For example, exchange reaction values of particular solutions can be used to predict metabolites that are consumed/produced by the organism which can be compared to available metabolomic data.^24^

Model standardization is commonly assessed through identifiers and annotations associated with model objects. Databases such as BiGG and ModelSEED set forth standardized identifiers for metabolites and reactions that allow for direct comparisons across models.^9,25^ The degree to which objects in the model correspond to these databases is used to assess model standardization. MEMOTE was developed by the metabolic modeling community in an effort to create a holistic quality assessment.^26^ MEMOTE conducts a uniform set of tests to assess both biological accuracy and model standardization then provides a detailed report with score insights and specific points of potential improvement. While benchmarking approaches are used to measure model quality, they also identify gaps in the model that serve as points for curation. Therefore, quality assessments are not the end point for the model development process, but rather a feedback mechanism to inform further curation.^27^

## Community metabolic modeling

**Fig. 2:**
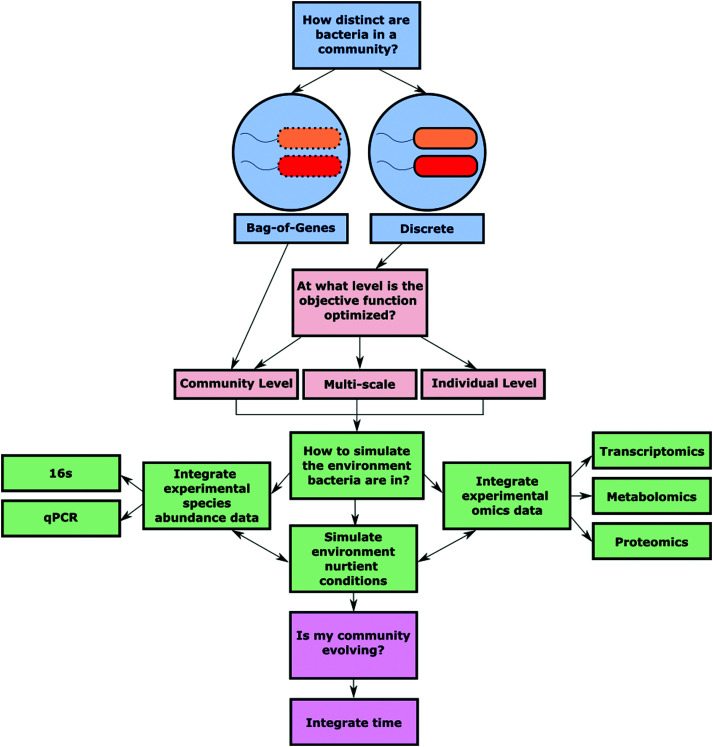
Essential decision points to consider during polymicrobial community model construction.

The foundation for quality analysis of a metabolic network lies in the completeness and accuracy of the GEM that is used for simulation. To make high-quality predictions about shared metabolites, community growth, and reaction flux, the starting bacterial GEMs should closely represent an individual strains’ metabolic capabilities. To aptly simulate polymicrobial metabolic nutrient cycling *in silico* a few key decisions must be made (Fig. 2).

### Fundamental assumptions

#### Compartmentalization

Once the initial models have been vetted and curated, the next step is determining how those models will simulate the interactions between bacteria. There are two primary options for accounting for how distinct bacteria are in a community: (1) the “bag-of-genes” approach or (2) the discrete approach.^20^ The bag-of-genes approach combines all the genetic makeup for the integrated models into one “super” bacterium.^28^ This approach has been used to construct metabolic models of microbial communities belonging to the human microbiome from metagenomic shotgun sequencing data.^29^ The authors explored community metabolic capabilities without the challenge of sorting reads into single-organism genomes. While such an analysis enables an interrogation of potential metabolic capabilities of a microbial community, this approach does not take into account the natural competition that occurs between bacteria as it allows for ubiquitous metabolite availability. The collective nature of the bag-of-genes approach does not consider species-specific transporters, which hinders the model's ability to accurately recapitulate physiological community interactions.

In the discrete approach, each bacterial model exists in a separate cytosolic compartment. Individual bacteria then interact metabolically through a shared extracellular space, where species-specific transport reactions are able to move communal metabolites into and out of the bacterial cytoplasm. This approach was used by Pacheco *et al.* to simulate the metabolic relationships that emerge when pairs of microbes are grown in co-culture.^30^ The models allowed for the identification of key cross-fed metabolites and how those metabolites impact the growth capability of each microbe. Spatially, this approach assumes that all bacteria have equal access to the communal metabolites. However, physiologically this equal access is often not the case. To simulate the uneven access to communal metabolites, Klitgord and Segrè incorporated another layer of compartmentalization *via* a shared environmental space.^31^ The shared environmental space allows for inter-species metabolite exchange *via* shuttle reactions, while individual extracellular compartments allow for bacteria-specific media conditions. Bacterial compartmentalization sets the stage for how an individual bacterial species will interact with other community members, as well as the surrounding environment. Thus, defining the spatial limitations of metabolite exchanges and metabolic machinery is a critical decision in any multi-species modeling effort.

#### Objective function

Choosing an objective function (OF) is the next defining feature for community metabolism modeling. There are three broad categories that encompass approaches for defining potential OFs: individual level, community level, or multi-scale. The goal of an individual-focused OF is to maximize an objective like biomass synthesis at the individual level, agnostic to the overall polymicrobial community biomass production. Community level OFs often optimize biomass production across all species, at the expense of the biomass production of some individual species. This assumption of “sacrifice” is counter to some observations, where bacterial guilds are selfish and prioritize individual biomass over community biomass.^32^ The argument is that bacteria have not necessarily evolved for optimization of a community biomass objective, even if participation in a community structure may be beneficial with respect to specific evolutionary objectives. Conversely, there are examples of bacteria participating metabolically in “social good” *via* individual sacrifice; however such cooperation and its associated mechanisms are only beginning to be understood.^32–34^

Multi-scale OFs often seek to optimize biomass production at both the individual and community level. Multi-scale OFs allow for the investigation of the trade-offs of bacteria sacrificing individual-level growth in exchange for greater community growth. These OFs strike a balance between the evolutionary concept that bacterial species focus on individual growth while also considering the evolutionary benefits of community-level metabolic interdependence. OptCom is a method that breaks down these two optimization equations into inner and outer problems.^35^ The inner problem is defined as individual-level biomass synthesis maximization, while the outer problem is community biomass synthesis maximization. The inner problem is optimized first in order to identify the maximum biomass production of each bacterial species. The outer problem is then optimized, but individual bacteria are allowed to grow at a proportion of their optimal biomass in order to simulate sacrifice of an individual species’ growth for community biomass synthesis maximization. The multi-scale tool MICOM offers the additional flexibility of manually adjusting the trade-off values assigned to individual bacteria, while also being able to integrate metagenomic community data.^36^

The definition of OFs for the simulation of a microbial community requires assumptions on global and local network optimization. Furthermore, how the objective function is defined influences what type of metabolic interactions a final model is best suited to investigate.

#### Time

FBA solutions offer insight into the instantaneous metabolic state of a given metabolic network. However, bacterial communities are in a continuous state of metabolic evolution. In order to simulate these dynamic events it is critical to account for temporal changes. Dynamic FBA (dFBA) is a dynamic modeling algorithm that incorporates time by iteratively calculating flux values and metabolite levels and using those values to update the physiological context of the model for the next simulated time point.^37^ The initial application of dFBA did not involve community modeling, and instead focused on the metabolic dynamics of an individual species. Dynamic multi-species metabolic modeling (DMMM) extends FBA to microbial communities by maximizing biomass synthesis of an individual bacterial species while continuing to account for community dynamics by tracking bacterial population sizes and metabolite concentrations.^38^ If an individual species is unable to produce maintenance levels of ATP, a death rate is calculated and applied to that species. The interplay of growth and death rates results in fluctuating population size, which directly affects the proportion of metabolites an individual species is producing and consuming, thus changing community metabolite composition. However, DMMM does not account for the metabolic changes that occur as a bacteria dies and its resources become available to the community. Additionally, DMMM lacks a clear option for integrating experimental data into the model.

Similarly, an extension of OptCom, dynamic-OptCom (d-OptCom) incorporates time by iteratively using the rates of community metabolism uptake and output, as the metabolic parameters for an individual bacteria's growth rate.^35,39^ With all dynamic models a primary drawback is the computational burden of running the analysis. DMMM circumvents this computational burden by solving FBA problems at a time point for each microbe. Brunner *et al.* reduced the number of optimization problems that have to be solved when running dFBA by using the solution from a previous time-step in dynamic analysis to inform the solution at the current time step, resulting in less computational power and time requirements.^40^

#### Space

Another key consideration for modeling polymicrobial communities is to incorporate a spatial component, which takes into account the constraints that physical location puts on a bacterial community. Accounting for the spatial aspect offers the ability to incorporate metabolic gradients that determine what metabolites individual bacteria can access. This metabolic gradient is dictated by bacterial production of metabolic byproducts and the spatial arrangement of all members in the community. COMETS is a tool developed to account for the spatial component of microbial communities, by integrating metabolite diffusion with FBA in order to evaluate how polymicrobial communities metabolically equilibrate.^41^ COMETS presents a unique opportunity to investigate how spatial considerations impact both community and individual level metabolism. Agent-based models (ABMs) allow for the integration of spatial considerations to investigate how an organism physically interacts with the surrounding environment. MatNet creates an intersection between GEMs and ABMs that allows for spatial considerations to be accounted for in metabolic network models.^42^ This approach was used to construct a multiscale model of *Pseudomonas aeruginosa* that was able to recapitulate decreased oxygen accessibility in relation to surface location in the context of biofilm formation. Similar to MatNet, BacArena is able to take into consideration the metabolic heterogeneity within a bacterial community due to its larger focus on an individual-centric ABM.^43^ BacArena was able to more accurately predict *Clostridium beijerinckii* doubling time when compared to COMETS. The number of tools available to study metabolic modeling with a spatio-temporal aspect is growing rapidly.^44,45^

### Construction

As for models of individual species, the quality of microbial community models can be enriched with the integration of experimental data. Since obligate commensal species are often difficult or impossible to culture in a laboratory setting, the ability of metagenomic, metatranscriptomic, metaproteomic, and metabolomic techniques to be used on naturally occurring communities makes these approaches exceedingly valuable. The collected data can be used to characterize the complex metabolic interactions occurring in microbial communities and can inform the construction, contextualization, and validation of community models.^46^

Once a microbial community is defined, the first step in a simulation is to characterize the members of that community. Metagenomic approaches such as amplicon-based sequencing are culture-independent methods that quantify species abundance in a community.^47,48^ While whole-metagenome sequencing (WMS), a type of amplicon-based sequencing, provides higher resolution data, revealing strain-level variation among community members, it is more expensive than lower resolution amplicon-based sequencing.^49^ These methods can be used in the investigation of communities that occur naturally or are difficult to culture. Metagenomic data can inform the decision of which individual models to include in a community simulation.^50^

### Contextualization

After the initial model has been constructed, more data can be integrated in order to better fit the model to a physiologically relevant context. Metagenomic profiling can be used to provide growth rate data for members of the community.^51^ In Descriptive OptCom, experimental growth rate data informs the biomass constraints for both individual bacterial species and the community.^35^ While this analysis provides useful insights into the metabolic activity of a community, it only offers a static snapshot. Metagenomics can also be performed across time points to provide dynamic abundance data to assess the growth of individual members of the community. Dynamic OptCom is capable of integrating this temporal data to investigate how the structure of a community evolves over time as individual members flourish or diminish.^39^ BOFdat integrates experimental growth data into the biomass objective function formulation of individual organisms, allowing for more accurate recapitulation of physiological metabolism.^52^ These individually curated GEMs can then be combined to simulate community interaction.

While metagenomics provides information about the microbes present in a community, communities of similar composition can still differ greatly in which functions are active.^53^ Metatranscriptomic and metaproteomic data characterize the RNA and proteins that mold community metabolism. Both of these data sets provide evidence for what metabolic pathways are being utilized by the community and therefore can be integrated as reaction weights.^54^ RIPTiDe and TIMBR are both integration algorithms that utilize transcriptomic data to create reaction weights, which then impact predicted model metabolic outputs.^17,55^ RIPTiDe additionally allows for this transcriptomic data to be used to “prune” reactions and metabolites from a model that do not have strong transcriptional support and are not necessary for an OF to carry flux. However, due to the varying residence time of RNA (∼3 minutes) and proteins (0.5–35 hours), these data offer different temporal snapshots of what is occurring in the organisms.^56^

Metabolomics can provide useful information about the shared metabolite pool and exchanges that occur in a microbial community.^39^ Exchange reaction constraints can be tailored to this data to reproduce metabolite availability, production, and consumption. Additionally, measured secreted metabolites can provide evidence about what pathways are active and can therefore be integrated as reaction weights.^21^

### Analysis

The analysis of community models often begins with validating *in silico* predictions with experimental data. Exchange reaction values from model simulations can be converted to metabolite abundances that can be compared to metabolomic data.^24^*In silico* growth of community members can be compared to abundance data from metagenomic profiling to evaluate accuracy of associated predictions. For example, Zuñiga *et al.* validated a community model by comparing simulated growth data to experimental bacterial community growth data.^57^ Additionally, Stolyar *et al.* built a metabolic model of methane production using a simple bacterial community composed of the sulfate-reducing bacteria *Desulfovibrio vulgaris* and the methanogen *Methanococcus maripaludis*.^58^ Their model was able to recapitulate experimental community growth data, while also differentiating between potential essential and nonessential interspecies electron shuttles for syntrophic growth. A validated model can then be used to explore bacterial relationships in different simulated contexts.

The contexts a model is best suited to investigate are inextricably linked to bacterial community construction (Table 1). A model that contains the genetic data for multiple bacteria can be used to identify essential genes in multiple contexts.^29^ Knowledge of identified essential genes can be utilized to design a single species that has the metabolic functionality of a larger community. Essential genes can also function as potential targets for novel drug development.^59^ Gene essentiality analysis allows us to identify how essential genes change after a selective pressure is applied to a community, resulting in the loss or mutation of previously essential genes over time.^60^ Cornerstone species in a community can be identified by finding the bacteria that provide the highest proportion of essential genes.^61^

**Table 1:** Comparison of key features that differentiate a subsect of polymicrobial community metabolic modeling algorithms

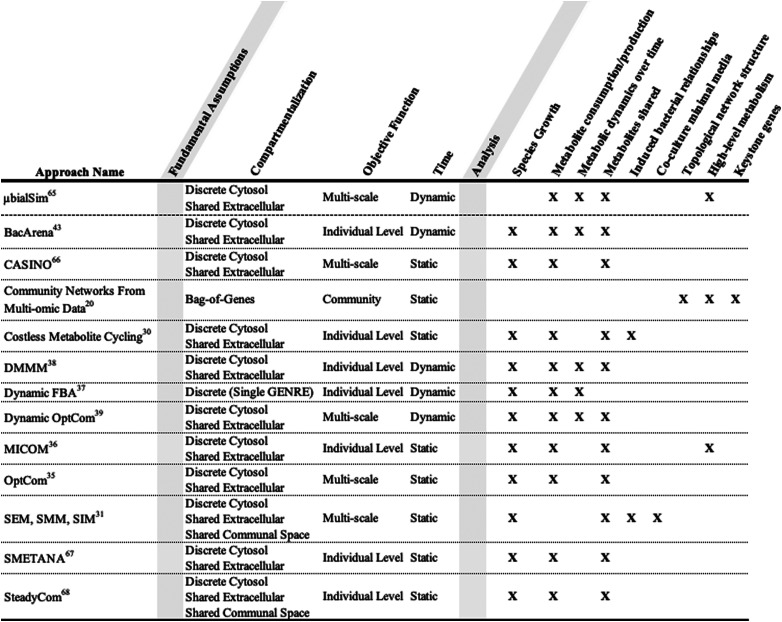

Metabolic relationships between bacteria can evolve naturally or be induced. Klitgord and Segrè developed three algorithms that allow for the identification of naturally occurring metabolic relationships, as well as the investigation of induced metabolic relationships (Table 1).^31^ SEM (search for exchanged metabolites) is a method to identify the minimal number of essential metabolites exchanged between bacteria for mutual growth in co-culture. SMM (search for minimal media) is an approach to create a minimal media for co-culture based on the essential metabolite exchanges. SIM (search for interaction inducing media) is a method to generate different media conditions based on which metabolites the respective bacteria are able to produce or consume. Predicted biomass synthesis from FBA simulations defines each media condition as having induced either neutral, mutual, or commensal interactions. In parallel with Klitgord's work regarding induced metabolic relationships, OptAux takes a genetic approach as opposed to a minimal media based approach.^62^ OptAux integrates time-course sequencing of co-cultured bacteria grown under forced cooperation conditions. OptAux utilizes this genetic data to make predictions regarding protein availability, and its impact on community composition.

Alternatively, Pacheco *et al.* treats the community setting as an “induction factor” in order to identify community-specific metabolite synthesis.^30^ The bacterial community growth is simulated on a set carbon source, and the metabolic byproducts of this growth are iteratively fed back into the community system as additional metabolic resources. The feedback loop runs until no new metabolites are produced, at which point the final community dynamic is assessed. While the metabolic composition of the polymicrobial community is iteratively updated, the population size of each bacterial species is not. Therefore, the model is limited in its ability to accurately represent metabolite concentrations due to the static nature of population sizes. Feasible experimental validation for induced metabolic cycling is severely limited by the ability to tag and observe cycled metabolites. In the context of the *Drosophila melanogaster* gut microbiome, Henriques *et al.* found that *Acetobacter pomorum* and *Lactobacillus plantarum* work in tandem *via* lactate cycling to overcome a deleterious host diet.^63^

Polymicrobial models can be used as a tool for optimizing community production of desirable metabolic byproducts through increasing overall community growth. Community flux balance analysis (cFBA) can be used to identify metabolically limiting factors that block increased community growth and therefore hinder the production of desirable products.^54^ Identifying context-specific metabolic limitations allows for potential circumvention *in vivo*, resulting in a more efficiently engineered bacterial community. However, the cFBA objective function works on the assumption that all bacteria in the community have symbiotic interactions. This assumption makes it more difficult to take into account resource competition and the “selfish” nature of bacteria, as well as potential mutualistic bacterial interactions, compared to other available tools.^32,38,64–68^

Conversely, Hibbing *et al.* modeled bacterial competition in order to explore how bacteria in a community system evolve to “cheat” the system.^64^ They discovered that for bacteria in the studied community it is more energetically favorable to “steal” thiamine from a neighbor than it is to produce thiamine itself. Nutrient cycling creates an environment that is conducive to the evolution of “lazy” bacteria, which over time will cut genetic “fat” in favor of scavenging the metabolic byproducts of its neighboring community members.^69^ However, if shared resources are in high demand, competitive selective pressures are likely to prevent a wide outcropping of “free-loading” bacterial populations. Thommes *et al.* explored how to intentionally design metabolically parsimonious bacterial communities through multi-strain *E. coli* computational models.^70^

An additional benefit of polymicrobial modeling is the relative ease with which alternative hypotheses can be explored.^71^ By simulating different single-carbon source utilization and implementing experimentally determined growth parameters, alternative metabolic pathways can be discovered and later experimentally validated. This approach can be extended to explore engineered polymicrobial systems. In some cases, potentially harmful metabolic byproducts are reduced or eliminated through co-culture consumption. One example is engineered *E. coli* to assist in carbon sequestration *via* an altered metabolism which allows for carbon dioxide to be converted to formic acid.^72^ Alternatively, polymicrobial modeling allows for the identification of critical metabolic interactions found in “harmful” bacterial interactions. If these keystone interactions are identified, they can subsequently be circumvented *via* antibiotic therapeutics that target specific bacterial metabolism. This type of exploration is especially relevant to human pathogenesis rooted in multi-bacterial infection. By excising the initial experimental step in favor of modeling, time and resources are saved, while a wide array of possibilities are explored.

## Future directions

Much like the bacteria they emulate, polymicrobial metabolic models are continuously evolving in regard to useful data integration approaches and relevant applications. These models offer inherent versatility that experimental methods do not possess. For example, models allow for the flexibility to simulate numerous conditions in which a polymicrobial community could be found. This ability is conducive to studying bacterial communities that may be hard to access, such as microbial communities in the human gut or anaerobes in inaccessible environmental niches like those found in deep sea vents, as well as bacterial communities that could be engineered and do not yet exist.^73^ Polymicrobial community modeling can quickly iterate through multiple potential growing conditions, and the subsequent output can be used to optimize desired metabolite production. Additionally, these simulations can be applied to systems that may be hard to isolate metabolically *in vivo*, such as host–pathogen interactions. Through simulating host–tissue metabolism in conjunction with a pathogen of interest, one can potentially observe how the infecting bacteria is metabolically altering the host for its own benefit.^74^ Such modeling efforts have the potential to drive much drug target discovery and the development of novel therapeutic strategies.

The interface of tissue and bacterial models can also be used to explore how metabolism functions in bacterial infections. For example, in the case of cystic fibrosis (CF), mucus hypersecretion creates a hospitable niche for bacterial growth, resulting in inflammation and hospitalization. Some species of bacteria, such as *Prevotella melaninogenica*, are known to degrade this mucus.^75^ Polymicrobial models can be used to simulate the metabolic interactions that occur as bacteria infect the lung epithelium. These models can offer a deeper understanding of how these infections occur, which can be used to improve therapies. Mucin catabolism in the context of CF is just one of many examples of how polymicrobial modeling can be applied to investigate novel therapeutic strategies. Polymicrobial models can also be used to identify metabolic links between primary and secondary infections, to elucidate how bacteria can work in chorus with one another.

The avenues of exploration that polymicrobial modeling present are limited by the number and quality of available models, as well as validated methods for identifying key genes, metabolites, and pathways important in specific clinical applications. However, with the continued development of more sophisticated methods and the improved quality of metabolic models, these constraints are rapidly dissolving.

## Conflicts of interest

There are no conflicts of interest to declare.

## Supplementary Material
